# For‐profit hospitals could play a distinctive role as anchor institutions

**DOI:** 10.1111/jep.13739

**Published:** 2022-07-19

**Authors:** Kelly Lynn Choyke, Berkeley Franz, Vanessa Rodriguez, Cory E. Cronin

**Affiliations:** ^1^ Heritage College of Osteopathic Medicine, Social Medicine Ohio University Athens Ohio USA; ^2^ College of Health Science and Professions, Social and Public Health Ohio University Athens Ohio USA; ^3^ The Appalachian Institute to Advance Health Equity Science Ohio University Athens USA

**Keywords:** anchor institutions, barriers, community engagement, for‐profit hospitals, population health

## Abstract

**Rationale:**

Hospitals have a longstanding presence in United States communities and contribute to economic development and community well‐being through widespread employment, purchasing and direct community engagement. Most of the data on anchor institutions to date, however, has focused on nonprofit organisations, especially nonprofit hospitals, colleges and universities. The aim of this study is to better understand if for‐profit hospitals engage in explicit anchor activities, and whether these organisations adopt unique strategies in carrying out this study.

**Methods:**

We used an inductive, qualitative approach to understand how for‐profit hospitals perceive their anchoring efforts as distinct as compared to nonprofits. We conducted in‐depth interviews with 23 hospital leaders, researchers and members of advocacy organisations, representing 11 different hospital organisations and 10 communities; and used thematic analysis to generate study findings.

**Results:**

For‐profit hospitals do see at least three primary differences that render them distinctive in their efforts to anchor themselves within their communities—namely, barriers that for‐profits encounter that nonprofits may not; their emphasis on strategic and synergistic practices; and their status as hospitals that also support their communities economically as tax‐paying entities.

**Conclusion:**

With a better understanding of their unique contributions as for‐profit organisations, policymakers can identify ways to leverage these hospitals to support their communities through outreach and engagement.

## 1 INTRODUCTION

Anchor institutions have an impact on the social, economic and physical well‐being of communities via their actions and interactions with citizens and other businesses, institutions and community stakeholders.^1^ Historically, anchor institutions have been defined as large organisations that serve as primary employers and purchasers in a community, indirectly impacting the economic health of the surrounding area. Recently, more expansive definitions have emerged that describe anchor institutions also in terms of their impact on community and individual well‐being through targeted outreach and engagement initiatives.^1^, ^2^, ^3^, ^4^, ^5^, ^6^ Examples of potential anchor institutions include large corporations and businesses, as well as medical and educational institutions such as hospitals, museums, libraries, colleges and universities that are unlikely to relocate due to their size and relationship with their community.^3^, ^7^, ^8^

Hospitals, as anchor institutions, are relied upon in their communities not only to keep citizens well; they also play a role as large employers and purchasers, as well as supporters of community health programmes and activities. The importance of this role is obvious in the distress experienced by rural areas and other underserved communities when hospitals face closure.^5^

In the United States, hospitals also vary considerably based on ownership type, which has important implications for anchor activities. The majority of hospitals in the United States are nonprofit, which means they are classified legally as 501(c)(3) charitable organisations. By law, this means that these organisations are exempt from a broad range of taxes; but must, in exchange, fulfil their charitable duties through providing reduced cost medical care to patients in need, and develop programmes to address critical health and economic needs in the community. This uniquely American model relies on private hospital organisations to fulfil the role that the government would typically play in providing social services and supporting community well‐being. In contrast, a growing minority of private hospitals in the United States are for‐profit and not legally obligated to be community‐engaged institutions.

While most research has been done on nonprofit institutions, for‐profit institutions also have the capacity to act as anchor institutions, both indirectly and directly, but may have different motivations for and approaches to doing so.^2^ This study aims to expand the understanding and categorisation of anchor institutions by considering how for‐profit hospitals might also participate in direct community engagement efforts, and whether these patterns of engagement are distinctive from nonprofit hospitals.

Businesses have long been recognized as vital to the communities they serve in terms of providing jobs, contributing to the local tax base, and supporting other businesses through supply procurement and other services rendered.^9^ But other contributions of large organisations, especially direct community engagement, have become increasingly vital, particularly in terms of healthcare, as state and federal retrenchment from community health over the past 60 years has opened up opportunities for private markets to meet the healthcare needs of communities across the United States. These changes have provided privately owned hospitals, both nonprofit and for‐profit organisations, with the opportunity to function as community‐engaged anchor institutions.

While academic debates over the differences between nonprofit and for‐profit hospitals have varied over the decades, much contemporary scholarship is in agreement that there are not substantial differences between nonprofit and for‐profit hospital systems in terms of mission, amount of uncompensated care provided, altruism, or institutional mortality rates.^10^, ^11^, ^12^, ^13^, ^14^, ^15^ Scholars have argued that while there are generalisable differences between nonprofit hospitals and for‐profit hospitals, such as the ways in which they are regulated, factors such as market mix, region, local health needs and demographics play a more significant role than ownership on the types and level of community health benefits and services.^16^, ^17^

Regardless of ownership status, hospitals are increasingly being urged to act as civically engaged members of their communities through their outreach and engagement efforts, as well as partnerships and sponsorships, in line with their role as significant investors, employers, customers and suppliers.^2^ This is, in part, due to positive community health models, embraced by most medical institutions, which emphasize interventions beyond traditional healthcare services, to impact social factors and ultimately downstream health outcomes. Proposed and newly implemented payment models^18^ are anticipated to incentivize hospitals to adopt community outreach activities; however, existing data are mostly limited to nonprofit hospitals. Because nonprofit, tax‐exempt organisations are subject to regular reporting guidelines overseen by the Internal Revenue Service, in exchange for 501(c)(3) status,^19^ we have considerable data on the activities they undertake to improve health and well‐being in their surrounding communities. Absent these same requirements, it is difficult to ascertain the contributions that for‐profit hospitals make, and whether ownership type is indeed related to hospital decisions to position themselves directly as community‐engaged anchor institutions.

The aim of this study, accordingly, is to explore how for‐profit hospitals perceive the role of anchor institutions—stable institutions that both provide employment and make other contributions to population or social health—in relation to their status as a corporate hospital. Our goal is to better understand the extent to which for‐profit hospitals perceive themselves as anchor institutions via their outreach and engagement efforts to improve community health. We utilize a qualitative approach to understand how for‐profit leadership weigh the importance of community engagement, and which perceived organisational factors, both internal and external, challenge and support this study. As such, we explore both direct efforts—community outreach and engagement efforts made with the express intent to positively impact individual and community well‐being; and indirect efforts—outreach and engagement efforts that may be more aligned with corporate responsibilities rather than social responsibilities (e.g., employee support programmes), yet have a positive impact on economic outcomes in the community. This study is the first to collect qualitative data on the engagement activities of for‐profit hospitals, and to assess how these organisations approach and perceive their role as community‐engaged anchors in relation to their corporate structure and fiduciary commitments. These data will provide a fuller understanding of the contributions of anchor hospitals and will inform both researchers and policymakers regarding how to properly track these activities going forward.

## 2 METHODS

The data for this analysis come from the Ohio University Anchors Study. The aim of this broader study was to understand how for‐profit hospitals perceive their levels of community engagement, and whether these actions are influenced by their ownership type. This project involved secondary quantitative data on the national landscape of for‐profit hospitals, and primary quantitative and qualitative data in the form of surveys and interviews with hospital leadership, staff and community residents. The research for this study included human subjects, and all participants provided informed consent. Approval was obtained from the Ohio University Institutional Review Board, and participants received a consent form and provided verbal consent before participating. No identifying information, personal medical information, or proprietary information was used in this study. The data for this study include qualitative, semistructured in‐depth interviews with hospital leaders and employees at for‐profit acute‐care hospital corporations and facilities in the United States. Because our goal was to understand how for‐profit hospitals perceive the roles they play in their communities, particularly their conscious or unconscious efforts to act as anchors, we used an inductive and interpretive qualitative strategy.^20^

### 2.1 Sample

The sample for this study includes twenty‐two representatives from four different for‐profit hospital corporations, and ten hospitals/hospital divisions located in the United States, as well as one representative from a for‐profit hospital interest group. For our research, we constructed a list of all acute‐care for‐profit hospitals in the United States, based upon data obtained from the 2015 American Hospital Association (AHA) hospital characteristics database, as well as enterprise level websites listing locations of acute‐care for‐profit hospital sites in the United States. All hospitals with current for‐profit status listed online or in the AHA database were eligible for participation in this study and were contacted for voluntary participation by a research team member via email and telephone. The researchers made an effort to recruit hospitals from across the United States and Puerto Rico, both rural and urban, as opposed to limiting the sample to one state or region, or only rural or urban locations; however, any acute‐care, for‐profit hospital that opted to participate was accepted into the study. Our goal was to include a diverse sample that, while not generalisable, would consider a range of circumstances. Of the twenty‐two representatives from for‐profit hospital corporations, twelve are representatives from ten individual hospital systems; eight representatives work within region‐specific multihospital systems (not at a specific hospital); and two work at the enterprise level of a for‐profit hospital corporation. Fourteen of the twenty‐three participants identify as female, while nine identify as male. Individual participant demographics are available in Table 1.

**Table 1:** Participant demographics as self‐reported during interviews, 2020

Participants	Gender	Level of employment	Hospital size by number of beds	Community	Role	Prior nonprofit and/or public employment
P1	F	Hospital	400+	Urban	Trauma programme director	Yes
P2	M	Corporate	N/A	N/A	Government affairs	Yes
P3	M	Hospital	1−49	Rural	CEO	Yes
P4	M	Hospital	1−49	Rural	COO	No
P5	M	Hospital	50−200	Urban	Physician liaison	No
P6	F	Hospital	200−399	Exurban	Community health	Yes
P7	F	Hospital	50‐200	Urban	CNO	No
P8	F	System	N/A	N/A	Communications	No
P9	M	System	N/A	N/A	Marketing	No
P10	M	Hospital	50−200	Urban	Physician relations	No
P11	F	Hospital	50−200	Urban	Director of strategy	No
P12	F	Corporate	N/A	N/A	Community engagement	Yes
P13	M	System	N/A	N/A	Marketing & community engagement	Yes
P14	F	System	N/A	N/A	Community engagement	Yes
P15	F	System	N/A	N/A	Communications & community strategy	No
P16	F	Hospital	200−399	Suburban	Marketing	Yes
P17	F	Hospital	200−399	Urban	Public relations & communications	No
P18	M	Interests Group	N/A	N/A	President & CEO	Yes
P19	F	Hospital	200−399	Urban	Business development	No
P20	F	Hospital	200−399	Urban	COO	No
P21	F	System	N/A	N/A	Division VP for strategic communications	Yes
P22	M	System	N/A	N/A	Director of community relations	Yes
P23	F	System	N/A	N/A	Director of community engagement	No

*Note*: Hospital‐one entity; System‐more than one hospital; Corporate‐all hospitals spanning various states or regions.

Participants are from hospitals (*N* = 11) presenting various combinations of the location, institution and community characteristics displayed in Table 2. All the hospitals are in counties with mixed nonprofit/for‐profit markets. Most hospitals are based in Southern or Western regions and, as defined by nonexistent state CON law programmes, in lax regulatory environments. Hospital capacity is diverse with a range between less than 49 beds to 400+ beds. The size of a hospital's system was likewise diverse with equal proportions of hospitals in systems with between 50 and 100 hospitals and systems with 100+ hospitals. Most of the hospitals are in urban locales, with the remainder of the hospitals split evenly between rural, suburban and exurban sites. Communities surrounding these hospitals were more likely to be racially diverse. Residents in these communities were divided evenly between the lower‐ and middle‐income categories, with 10% in the highest‐income category.

**Table 2:** Hospital Characteristics (*N* = 11)

Location			
County market	US region	Regulatory environment	
Mixed = 11 (100%) NP = 0 (0%) FP = 0 (0%)	Northeast = 1 (10%) South = 6 (50%) Midwest = 0 (0%) West = 4 (40%)	Strict = 2 (18%) Lax = 9 (81%)	

Institution			
Beds	System size by hospitals	Designation	Change in status
1−49 = 2 (18%) 50−200 = 4 (37%) 201−399 = 3 (27%) +400 = 2 (18%)	0−49 = 2 (18%) 50−100 = 4 (37%) +100 = 5 (45%)	Sole community provider = 1 (9%) Critical access = 1 (9%)	NP to FP = 1 (9%) Public to FP = 0 (0%)

Community			
Type	Racial demographics	Median household income	
Urban = 8 (73%) Exurban = 1 (9%) Rural = 1 (9%) Suburban = 1 (9%)	Diverse = 7 (63%) Homogenous = 4 (37%)	$0−$44,999 = 5 (45%) $45,000−$149,000 = 5 (45%) $150,000 = 1 (10%)	

*Note*: FP, for‐profit; NP, non‐profit.

### 2.2 Data collection

Individual acute‐care for‐profit hospitals, as well as hospital systems and hospital interest groups were contacted by email and phone to request participation in this study. Data were collected between March 2020 and April 2021. Once employees were identified for participation, and consent was received, a phone or web interview was scheduled. If, in the initial interview, some of our questions or topics of discussion were not able to be addressed, an additional interview was requested with an employee who could provide the missing information. Hospital leaders or employees with knowledge of outreach and engagement efforts were requested for interviews. We are confident we interviewed the people best suited to speak regarding the hospital's overall outreach and engagement efforts, as they were either hospital leaders or employees involved in the day‐to‐day implementation of such efforts. The interviews were led by a doctoral‐trained team member with expertise in qualitative and ethnographic methods and mass communications. The interviewees were asked questions pertaining to outreach and engagement efforts, and perceived barriers—both internal and external—to such; anchor roles and activities, and willingness to report aforesaid efforts to the public. Interviews were audio recorded and professionally transcribed for data analysis.

### 2.3 Data analysis

The interviews were coded by a team member trained in qualitative and ethnographic data collection and analysis, and mass communications, utilising the qualitative data analysis programme, Dedoose,^21^ while employing approaches drawn from constructivist grounded theory analysis.^20^ Within this foundation, concurrent data collection and data analysis take place. This means interviews are coded directly after they are conducted; and preliminary findings are then used to guide forthcoming interviews and adjust the interview guide. Coding occurred in three stages, including line‐by‐line and section‐by‐section coding, secondary coding to highlight developing categories, followed by the categorisation of themes that emerged from codes. The entire research team wrote memos throughout the coding process, and met weekly to discuss the emerging themes. The final product presented in this manuscript is a set of themes related to how for‐profit hospitals understand their anchor activities, and position within the larger healthcare landscape in the United States.

## 3 RESULTS

Interviews with for‐profit hospital leadership suggest that these organisations prioritize community engagement and approach the role of anchor institution from a unique perspective as for‐profit hospitals. Hospital leaders suggested that for‐profit organisations may face unique external and internal barriers in enacting community engagement, are able to align their contributions with their corporate strategy, and make additional contributions as tax paying entities. All of the hospital employees that participated in this study viewed their hospitals, or hospital systems, as anchors within their communities. Not all participants were aware of the terminology at first, but once the definition of an anchor institution was explained, they exuberantly declared their institution to be a community anchor: their hospital(s) provide employment, healthcare, outreach and engagement, and regularly partner with and support other community stakeholders, businesses and charities. To be clear, while some hospitals and healthcare institutions are aware of anchoring philosophies, it is not uncommon for healthcare professionals or healthcare researchers to be unfamiliar with such terminology, as the use of the term anchor is not widespread in professional or academic spaces. Although hospitals and hospital employees may not be familiar with the terminology does not mean their institution has not been engaging in anchor activities. Regardless of terminology, all participants were able to verbalize and justify why they perceived their institution to be involved in anchor activities.

For example, after explaining the meaning of an anchor institution to a participant working in community engagement, one hospital responded, ‘I'd have to say we are an anchor. We definitely have been in this community for over 150 years. We do employ most of our local communities as healthcare personnel here at the hospital’ (P6). Other participants were well aware of the designation of anchor institutions, and have always seen their hospital, or hospital systems, as anchors. A systems level employee in marketing and community engagement declared, ‘I mean, the whole point of an anchor is that you're stable, and we've been here for 75 years, and we're the oldest hospital in this area…’ (P14). Another participant working in marketing and community engagement added, ‘So, I think we are, from an employment standpoint, anchor institutions. Certainly, the taxes we pay help to fund our schools, our firefighters, our police and those kinds of services. Our charitable partnerships and charitable giving contribute to it, and the help‐oriented work we do with the community, whether it be health education, screenings, et cetera, I think contribute to it as well’ (P13). Other leaders agreed, arguing that their institution made important contributions to the community through taxes paid, employment, and the provision of community health services.

**1**: Perceived Barriers

Beyond serving as stable employers and purchasers in their communities, participants also emphasized efforts toward community engagement, as well as significant barriers that limited the impact of this study, due to their ownership type. Participants suggested both perceived external and internal barriers to for‐profit hospitals serving as community‐engaged anchor institutions, which included regulatory barriers, less staff, fewer funds for external communications, and negative public perceptions of for‐profit hospitals compared to their nonprofit counterparts. Regulatory barriers vary by state; however, lack of access to programmes such as 340B was cited as a universal point of contention among for‐profit hospitals, particularly in the context of serving as engaged community partners. 340B is a Medicaid Drug Rebate Programme enacted in 1992 that requires pharmaceutical manufacturers to provide rebates or discounts to healthcare organisations supplying Medicaid recipients with outpatient pharmaceuticals.^22^ As a leader at a for‐profit interest groups explained, ‘You can look at the particulars at some big teaching hospitals and their margin…Some incredibly high percentage of their margin actually is the savings they're otherwise getting from 340B, and the competitive edge they get from it’ (P18). For‐profit hospitals provide similar amounts of care for Medicaid, Medicare and uninsured patients as nonprofits.^14^, ^23^ Thus, being excluded from 340B due to ownership type was cited as a particular point of frustration. As an employee at a for‐profit hospital system in governmental affairs explained:

Honestly, every single thing that a nonprofit does, we do. The only difference, the only distinction that I can think of from a public policy that we don't have access to that nonprofits do is something called a 340B Direct programme…. And that is reduced priced drugs, which is kind of a controversial programme, but it goes to nonprofit hospital outpatient facilities. We don't have access to that, which is ludicrous because, again, we take the same number if not more uncompensated care, and if not more Medicaid, at least the same. But anyway, that's the only functional difference in terms of any sort of public policy that impacts the community (P2).

As participants explained, not having access to subsidies like 340B means that there are fewer discretionary dollars to put back into the community as part of direct engagement efforts. Although nonprofit and for‐profit organisations both have the potential to passively anchor through employment efforts and purchasing, for‐profit hospital participants felt that policies still favour nonprofit hospitals, benefitting them with more financial leverage to offer direct community engagement programming as anchor institutions.

Additional barriers included restrictions that inhibit for‐profit hospitals from collecting and distributing charitable funds, which may limit the amount of community engagement that for‐profit hospitals can undertake. However, this does not prevent for‐profit hospitals from anchoring; these restrictions simply limit resources dedicated to charitable programmes. Several for‐profit leaders noted that charitable giving and receiving can be more complex for for‐profit organisations, and that they feel obligated to comply with a range of regulations and limitations that they described as not applying to nonprofit organisations. As a physician liaison at a hospital stated, ‘We have to be very careful with how we help with fundraisers, just because of the different laws with what we can contribute, and how we contribute to certain things’ (P5). Furthermore, a hospital employee in community health explained that lack of access to grants and foundational funds limited outreach and engagement efforts: ‘As a for‐profit, it's hard because I just don't have the opportunity for grants’ (P6). For employees whose work directly relates to community health improvement, they felt the inability of for‐profits to source capital for outreach and engagement via direct charitable contributions, gift‐giving, and state and federal grants was a significant external barrier; while such barriers do not prevent for‐profit hospitals from anchoring, they restrict resources and opportunities for community engagement.

In addition to external barriers, when for‐profit hospitals undertake charitable activities, they also have less staff to communicate the scope and impact of these activities, which is an internal barrier resulting from the organisational structure and practices of for‐profit hospitals. As one participant working in communications explained, ‘In the for‐profit world, you have a lot fewer people that are staffing things. And I don't mean on the clinical floor, staffing patients, but I mean in roles such as mine or others’ (P15). Nonprofit hospitals tend to have more staff members in administrative positions, and this was perceived as a significant internal barrier to communicating for‐profit anchoring narratives, or altruistic stories, to the public. Factors inhibiting for‐profit hospitals from externally communicating about their anchoring efforts more directly and abundantly were considered key internal barriers because this compounds what for‐profit hospitals believe to be negative public perceptions regarding their business model. Participants discussed distrust of for‐profit hospitals as one of the primary barriers specific to for‐profit hospitals, as they must battle assumptions about why they provide care and, consequently, the quality of the care they provide based upon their revenue structure. As a director of community engagement explained:

But then also, another barrier that I have seen is that a lot of organisations are hesitant to partner with us because the way they might be perceived out in the community for supporting a for‐profit healthcare system as part of a programme or initiative versus a not‐for‐profit hospital. That's something that I have experienced and noticed things over the years, is that we just carry that stigma out there with a lot of people (P22).

As participants explained, stigma related to being a for‐profit hospital often limited partnerships and, thus, the ability to participate in community engagement.

**2**: Corporate Strategy

Hospitals are community stakeholders, and they rely on revenue and goodwill in the community to operate successfully. Given the corporate nature of for‐profit hospitals, this is especially true for these organisations, and means that anchor activities are intertwined with their business model. In other words, corporate responsibility coincides heavily with social responsibility in the operation of for‐profit hospitals. Most for‐profit hospitals are also members of larger multihospital and investor‐owned or publicly traded companies; thus, ROI (return on investment) is an important factor considered in making anchoring decisions. While participants did not report that leadership inhibited outreach and engagement efforts in any way due to desires to increase revenue for ROI, participants deployed corporate strategies, or best practices, to outreach and engagement efforts to maximize use of resources as well as the results of anchoring efforts. As one participant explained:

So, we do have to ask ourselves, ‘Does it serve our patients in our geographic areas that we serve? Does this align to our goals and to what we do as a hospital?’… I think as resources become more limited, hospitals may want to see more of an ROI on their investments…So I would look and give priority to events that enable us to engage with people more, to have a deeper connection with people (P17).

Participants also suggested that strategic sponsorships, partnerships, and outreach and engagement in the local community were good for institutional branding. In other words, getting the name of the hospital out there and fostering a positive perception of the organisation was important. Participants also explained the impact of sharing the unique contributions of their organisation to distinguish it not only as an anchor institution, but as one that offers corporate expertise. One employee working in outreach explained that, ‘We want to work in the community in areas where we have something to offer that's unique. So that would be areas where we have business expertise, areas where we have knowledge or data, areas where we could really lean in and make a difference’ (P12). Of course, for‐profit hospitals are also still expected to do the usual things that hospitals do, including providing quality services to meet community needs and providing uncompensated care in the emergency department. But in distinguishing themselves as anchor institutions, for‐profit leadership argued that the contributions of their organisations were not only vital to their communities, but there was value‐added from their particular organisational configuration as tax‐paying entities that are strategic about outreach and engagement efforts.

**3**: Tax‐Paying Entities

When asked how participants understand how for‐profit hospitals may be different, or unique as anchor institutions, their status as tax‐paying entities stood out as a primary characteristic of how for‐profit hospitals have a unique impact. Participants were quick to point out their hospital, or hospital system, behaved as stable anchors, providing employment, healthcare, outreach and engagement, but also contributing additional economic support for their communities and surrounding areas due their status as tax‐paying hospitals. For example, a hospital employee in marketing and public relations commented, ‘But the part that I don't think always gets told is, as a for‐profit hospital, the taxes that we pay that come back to the community’ (P16). In addition to the anchor activities shared by most hospitals, the taxes that for‐profit hospitals pay fund the education system, police, fire and rescue, transportation, and parks and recreation, among other services. ‘But those taxes pay for schools, they pay for bus drivers, they pay for people to pick up your trash. They pay for the infrastructure of the city’, argued a hospital employee working in community engagement (P12). Another hospital employee working in community engagement pointed out that being a tax‐paying entity is the primary difference between for‐profit and nonprofit hospitals:

…for‐profit institutions, hospitals, pay taxes. We pay property taxes, unlike our nonprofit friends in the healthcare industry. So, that is obviously a huge amount of money that we are paying and putting back into the cities that we serve. So, that's probably one of the distinct differences between for‐profits and nonprofits. That, and the fact, of course, that nonprofits can fundraise for their activities, and the investor‐owneds don't have that opportunity’ (P23).

Hospitals, regardless of ownership type, are often one of the primary employers and sources of revenue for communities; and when those hospitals are also tax paying entities, they have the potential to contribute additional economic resources to fund the infrastructure and necessary social programmes of that community. Thus, for‐profit hospital leaders saw their organisations as unique anchors in the hospital market because they fulfil their hospital mission and community expectations of hospitals, regardless of ownership, while also paying taxes. The contributions of the tax‐paying hospital, in other words, cannot be understood without acknowledging the contributions that for‐profit hospitals make to communities looking for relief from financial burdens. Such communities may be in need of the taxes that a for‐profit hospital would provide, in addition to the traditional services offered by a community hospital. Participants argued, accordingly, that for‐profit and nonprofit hospitals generally share the same missions and provide similar services and levels of uncompensated care, but that for‐profits also make an important contribution to the local tax base. Participants did not imply that for‐profit hospitals are superior anchor institutions simply because they pay additional taxes. However, the additional economic support that communities receive from tax paying hospitals is a significant factor in understanding the characteristics that distinguish for‐profit hospitals as unique anchor institutions.

## 4 DISCUSSION

The regulatory requirements placed upon nonprofit hospitals, and the tax exemption received, have generally drawn greater attention to these organisations as compared to their for‐profit peers in the context of community engagement and economic impact. The lack of readily available data on for‐profit hospitals has exacerbated this gap in knowledge of the community engagement activities these organisations undertake. By talking directly to leaders of these organisations, we are better able to understand how these organisations view themselves in the broader context of anchor hospitals, and how they compare themselves to hospitals of other ownership types.

In line with previous literature, these conversations indicate that for‐profit hospitals do engage in the types of activities that are legally characterized as hospital community benefit, even though they are not required to do so. Additionally, for‐profit hospitals are active in programming that would be considered a part of an anchor organisation's mission and in alignment with the definition of an anchor institution.^10^, ^23^ Leaders of these organisations also provided insight into how they view their tax‐paying status. They felt that, unlike their nonprofit counterparts, their hospitals contribute to their communities through tax revenues at the same time that they offer programming and community enrichment, ultimately compounding their community contributions.

Our work suggests that for‐profit hospitals look strategically at the things they can do in their community, and as such, there is the potential for for‐profit hospitals to make unique contributions as anchor institutions. There are common efforts undertaken by hospitals generally, such as uncompensated care; but beyond this, we find that for‐profit hospitals tend to seek out investments that are mutually beneficial for the community and the hospital, as well as efforts for which they will receive recognition. For example, our findings show that for‐profit hospitals place importance upon being viewed as good community stewards and partners. In carrying out anchor activities, these organisations appear to emphasize a potential return on investment.

Beyond adopting strategic anchor initiatives, hospital leaders suggested that key internal and external barriers exist that may limit the impact of for‐profit hospitals' work. Often the best strategy to addressing community needs is to invest in or partner with another organisation more directly involved with the provision of social services to address local issues. However, our conversations illuminated that for‐profit hospital leaders perceived barriers to some of these partnerships, due to their ownership status. These leaders expressed a feeling of bias because of their for‐profit status, stating that no matter the intention of community investments, there exists an assumption that their profit motive drives all decisions, even their primary mission as a hospital. In discussing communication, for‐profit hospital leaders expressed that they felt nonprofit organisations had a higher need to communicate their contributions to the community to justify their tax‐exempt status, and therefore fostered more goodwill in the community. Furthermore, participants discussed lack of access to grants and fundraising; in particular, for‐profits are largely excluded from fundraising, charitable gifts and donations, and grants.^24^ Though multi‐hospital system membership is becoming increasingly common for both nonprofit and for‐profit hospitals, for‐profit hospitals are disproportionately affected by this trend. Centralized decision‐making, removed from the local community, may also affect the resources dedicated to community efforts and the strategies a hospital employs.

Overall, the nature of a for‐profit organisation may present both unique opportunities and barriers to hospital‐community engagement. These findings add to the existing literature on profit status and community engagement by sharing the perspectives of leaders from for‐profit organisations.^10^, ^23^, ^25^, ^26^ At a minimum, for‐profit hospitals may fulfil at least part of the definition of an anchor institution through their existence and their roles as an employer, purchaser and source of tax revenue. However, decision‐makers within for‐profit hospitals report intentionally going beyond this and participating in direct community engagement to improve health and well‐being.

### 4.1 Limitations

This study has limitations, the most notable of which is its reliance on self‐reported and self‐described information. Self‐reported data provides considerable strengths given the exploratory research question regarding perceptions of community engagement efforts, but is also prone to social desirability bias. Because for‐profit hospitals are not required to report on their community benefit activity, there is no way to independently assess or analyse the activities discussed by our participants. Nonetheless, this approach allowed us to gather rich data on the anchor initiatives of for‐profit hospitals, and identify key ways in which they may be distinctive as anchor institutions. Because nonprofit hospitals were not included in this study, however, we are unable to assess whether nonprofit hospitals may also experience similar perceived barriers. Future work should extend these findings to a broader group of for‐profit and nonprofit hospitals, and explore why these organisations undertake anchor activities above and beyond those that are legally required, and whether different patterns of investment and programmes are evident. Future studies should also identify ways to measure the tax‐related and other financial contributions that for‐profit versus nonprofit hospitals make to communities, and their associated impact on economic and health outcomes.

## 5 CONCLUSION

For‐profit hospital organisations are unique systems. Our study is the first to assess whether hospital leaders perceive their organisational structure to have a role in their work as community‐engaged anchor institutions. Although for‐profit hospitals have similar missions to nonprofit hospitals, in the United States they exist in a different regulatory atmosphere than their nonprofit counterparts. As a result, these differences seem to shape for‐profit hospitals' approaches to community engagement, and create internal and external barriers unique to for‐profit systems. Due to the differing ownership and regulatory structures, hospital leaders suggested that for‐profit hospitals focus on strategic practices in their partnerships, relationships with their communities, and their outreach and engagement practices in an effort to maximize the return on their services, both to the public and their investors. With a better understanding of their unique contributions as for‐profit organisations, policymakers should identify ways to leverage these hospitals to participate in community engagement and development initiatives.

## AUTHOR CONTRIBUTIONS

We verify that Kelly Choyke, Berkeley Franz, Cory Cronin and Vanessa Rodriguez have substantially contributed to the paper. Please see contributions for each author below. **Kelly Lynn Choyke**: Conceptualisation, data curation, formal analysis, investigation, methodology, project administration, resources and writing—original and review and editing. **Berkeley Franz**: Conceptualisation, data curation, formal analysis, investigation, methodology, project administration, resources, supervision, project administration, funding acquisition, visualisation and writing—original and review and editing. **Vanessa Rodriguez**: Data curation, investigation and resources. **Cory Edward Cronin**: Conceptualisation, data curation, investigation, project administration, resources, supervision, project administration, funding acquisition, visualisation and writing—original and review and editing.

## CONFLICT OF INTEREST

The authors declare no conflict of interest.

## ETHICS STATEMENT

The research for this study includes human subjects, and all participants provided informed consent. Approval was obtained from Ohio University's Institutional Review Board, IRB number: 19‐x‐198, and participants received a consent form and provided verbal consent before participating. No identifying information, personal medical information, or proprietary information is used in this study.

## Data Availability

Data are available upon reasonable request to the authors.
